# Serum Amyloid A and Haptoglobin as Markers in Cats with Gingivitis—Preliminary Study

**DOI:** 10.1002/vms3.70606

**Published:** 2025-09-13

**Authors:** Ioannis L. Oikonomidis, Ioannis Kavarnos, Serafeim Papadimitriou, Jose Joaquin Ceron, Maria Kouki, Katerina K. Adamama‐Moraitou, Nectarios Soubasis

**Affiliations:** ^1^ Department of Veterinary Anatomy Physiology and Pathology Institute of Infection Veterinary and Ecological Sciences University of Liverpool Neston UK; ^2^ Companion Animal Clinic School of Veterinary Medicine Aristotle University of Thessaloniki Thessaloniki Greece; ^3^ Interdisciplinar Laboratory of Clinical Analysis University of Murcia Murcia Spain

**Keywords:** acute phase proteins, feline, inflammation, periodontal disease, SAA

## Abstract

**Background:**

Chronic gingivostomatitis has been associated with increases in α1‐acid glycoprotein and serum haptoglobin (Hp) in cats. However, serum amyloid A (SAA) and Hp have not been previously evaluated in cats with uncomplicated gingivitis.

**Objectives:**

To compare SAA and Hp between cats with gingivitis and healthy cats, and to investigate the correlation between these two proteins and the severity of gingivitis.

**Methods:**

This was a prospective cross‐sectional study. Adult, FIV‐ and FeLV‐seronegative cats were included. The cats were allocated into two age‐ and sex‐matched groups. The case group included cats with gingivitis, and the control group included clinically and clinicopathologically healthy cats. The severity of gingivitis was assessed by the Total Mouth Periodontal Score (TMPS)‐G index. Serum samples were used to measure SAA and Hp using a previously validated turbidimetric immunoassay and haemoglobin‐binding method, respectively. The R statistical language was used for the statistical analysis.

**Results:**

A total of 22 cats were included, 11 in each study group. The median (range) age of cats was 5.0 (3.0–11.0) years. The median Hp concentration was significantly higher (*p* = 0.001) in the case group (2.40 [0.72–4.44] g/L) compared with the control group (1.06 [0.50–1.42] g/L). A significant correlation was found between Hp and TMPS‐G (rho = 0.636, *p* = 0.040). The SAA was below the detection limit (0.4 mg/L) in all samples of the control group and in 10/11 samples of the case group.

**Conclusions:**

Feline gingivitis is associated with increased Hp, suggesting the presence of an acute‐phase reaction. Haptoglobin appears to be correlated with the severity of the disease.

## Graphical abstract

• Haptoglobin concentration was significantly increased in cats with gingivitis and correlated with disease severity, suggesting its potential as a biomarker.

• In contrast, serum amyloid A was undetectable in most cases, indicating limited utility in feline gingivitis.

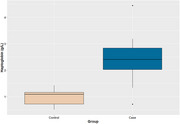

## 1 Introduction

Periodontal disease is a group of conditions characterised by plaque‐induced inflammation of the periodontal tissues. In a study which involved more than 15,000 cats admitted to different veterinary practices, dental disease was the most commonly reported disease. Specifically, calculus and gingivitis were present in 24% and 13% of feline patients, respectively (Lund et al. 1999). In another recent study, periodontal disease had a 1 year period prevalence of 15.2% in cats under primary veterinary care in the United Kingdom (O'Neill et al. 2023).

Periodontal disease is initiated when bacteria, in the form of plaque, adhere to the tooth. If plaque is not removed, gingivitis may occur within 2–3 weeks. Damage to the junctional epithelial cells that form the barrier at the gingival attachment to the tooth may allow bacteria and bacterial products, including cytotoxins and endotoxins, to enter the connective tissue beneath the epithelium (Dentino et al. 2013; Perry and Tutt 2015). The host immune response to these bacteria and bacterial products, which is triggered in order to eliminate the threat, dictates whether gingivitis will resolve or progress to a state of chronic inflammation, resulting in further destruction of the soft and hard periodontal tissues (Rossa and Kirkwood 2012).

The acute phase response or reaction, refers to a non‐specific, complex reaction that occurs shortly after an inflammatory stimulus as a part of the innate defence system of the organism (Cerón et al. 2005). The hallmarks of acute phase response are three: fever, leucocytosis and alterations in the concentration of serum acute phase proteins (APPs) (Paltrinieri 2008). The APPs can be classified as positive (whose serum concentration increases) or negative (whose serum concentration decreases). The concentration of positive APPs starts to increase within a few hours of the inflammatory stimulus, peaks in 24–48 h and remains increased for as long as the inflammatory stimulus persists (Paltrinieri 2008). Therefore, positive APPs are used for early detection and potentially monitoring of inflammatory diseases. The positive APPs can be further classified as major or minor based on the magnitude of the increase, using a 10‐fold increase as a cut‐off (Cerón et al. 2005). Based on the available scarce literature on feline APPs, serum amyloid A (SAA) and α1‐acid glycoprotein are considered major APPs, while haptoglobin is considered a minor APP (Petersen et al. 2004).

Periodontal disease has been associated with acute phase reaction in both humans (Ebersole et al. 1997) and dogs (Kouki 2013; Rawlinson et al. 2011). To our knowledge, there are only two published studies on APPs in cats with periodontal disease (Mestrinho et al. 2020; Polkowska et al. 2018). Both studies included cats with chronic gingivostomatitis and found elevations in serum α1‐acid glycoprotein (Mestrinho et al. 2020) and haptoglobin concentrations (Polkowska et al. 2018) as compared to healthy cats.

To our knowledge, the APPs have not been previously evaluated in cats with gingivitis without evidence of progression to periodontitis and/or stomatitis. Therefore, the aims of our study were to compare the SAA and haptoglobin concentrations between cats with gingivitis and healthy individuals and to evaluate the correlation of these APPs with the severity of gingivitis. We hypothesised that the concentration of SAA and haptoglobin would be significantly higher in cats with gingivitis compared to healthy cats, and that the two proteins would be positively correlated with the severity of gingivitis.

## 2 Materials and Methods

The present study had a prospective cross‐sectional design and was performed at a veterinary teaching hospital. The cats included in this study were allocated into two age‐ and sex‐matched groups. The case group included cats with gingivitis, and the control group included healthy cats without any evidence of gingivitis. The inclusion criteria that should be met for cats included in the case group were as follows: (i) age > 1 year; (ii) fasting for 12 h; (iii) no medication or history of illness during the preceding month; (iv) unremarkable serum biochemistry; (v) clinical evidence of gingivitis; and (vi) seronegativity for FIV antibodies and FeLV antigen (SNAP FIV/FeLV Combo Test, IDEXX Laboratories, USA). Cats with gingivitis and an underlying disease were excluded. The inclusion criteria that should be met for cats included in the control group were as follows: (i) age > 1 year; (ii) fasting for 12 h; (iii) no medication or history of illness during the preceding month; (iv) unremarkable physical examination, full blood count and serum biochemistry; and (v) seronegativity for FIV antibodies and FeLV antigen (SNAP FIV/FeLV Combo Test, IDEXX Laboratories, USA). Age matching was performed by identifying a healthy individual of the same age from the pool of eligible controls. This one‐to‐one matching ensured that age distributions were comparable between the groups and minimised age‐related confounding in the analysis. Informed consent was obtained from all participants’ owners.

In order to determine the intensity and the extent of periodontal disease, the Total Mouth Periodontal Score (TMPS) system was used (Harvey et al. 2008). This system allows an accurate, repeatable measure of the full extent of periodontal disease in a particular patient at a particular time point by using a blunt‐tipped calibrated periodontal probe. The measurements were taken from the circumference of every tooth present inside the oral cavity as soon as the cat was anaesthetised and before any other procedures. The measurements were inserted in a spreadsheet, and two indices were automatically calculated for the gingival bleeding and periodontal destruction (TMPS‐G and TMPS‐P, respectively).

These indices represent the intensity of gingival inflammation (TMPS‐G), by means of the Gingival Bleeding Index (GBI) and extent of periodontal destruction or attachment loss (TMPS‐P) (Harvey et al. 2008). The measurements were always recorded by two trained examiners (SP, MK). The GBI was recorded for the buccal and palatal/lingual surface of each tooth as follows: 0 = no inflammation: normal gingiva; 1 = mild inflammation: slight change in colour, slight oedema, no bleeding on probing; 2 = moderate inflammation: redness, oedema, glazing of surface, bleeding on probing within 30 s; 3 = severe inflammation: spontaneous bleeding or immediate bleeding on probing.

The periodontal destruction, if any, was graded based on the probing depth, defined as the greatest distance (mm) from the cementoenamel junction to the bottom of the pocket. Measurements were taken from all roots by placing the probe close to parallel to the long axis of the root. Only pockets > 0.5 mm in depth were recorded in the spreadsheet. The TMPS spreadsheet for cats was made available by C.E. Harvey on request.

The time required for diagnostic procedures was approximately half an hour, which included routine intraoral radiography and measurements performed at our institution. The total duration of anaesthesia varied depending on the specific purpose for which each animal was anaesthetised. However, all animals participating in the study underwent elective or minor dental procedures, such as professional scaling. Generally, the duration of general anaesthesia and recovery did not exceed 2 h.

The anaesthetic protocol was identical for all cats: after a 12 h fasting period, acepromazine maleate (0.05 mg/kg) with butorphanol (Dolorex, MSDUK) (0.1 mg/kg) IM was used for premedication. General anaesthesia was induced with intravenous propofol 2% (Propofol, Braun AG, Melsungen, Germany) 4 mg/kg and maintained with isoflurane (Isoflurane, Baxter, Allerød, Denmark) delivered in oxygen.

The samples used in this study were aliquots of blood samples collected for diagnostic purposes. Blood samples were collected via jugular venepuncture into plain tubes (Deltalab, Spain). The blood samples were allowed to clot for 20 min and were centrifuged at 1800 g for 5 min. The harvested serum samples were placed in plain tubes and stored at −80°C for 6 months prior to analysis. Samples with macroscopically evident haemolysis or lipaemia were excluded from further analysis.

SAA concentrations were determined by a human turbidimetric immunoassay (LZ‐SAA; Eiken Chemical Co.) that was previously validated for use in cats (Hansen et al. 2006). Serum haptoglobin concentrations were determined by the haemoglobin‐binding method with the use of a commercial kit (Tridelta Development Ltd.). The method was previously validated for use in cats (Tvarijonaviciute et al. 2012).

The sample size was calculated to achieve a statistical power of 0.8, with a significance level set at 0.05. It was assumed that the control group would have a mean haptoglobin concentration of 1.14 g/L and a standard deviation (SD) of 0.29 g/L, while the case group would have a mean of 2.24 g/L. The control group's mean and SD, as well as the SD of the case group, were extrapolated from the study of Polkowska et al. (2018), while the mean haptoglobin for the case group was set at one‐third of the value reported for cats with gingivostomatitis in the same study. The data distribution was evaluated using the Shapiro–Wilk test. Median comparisons between the two groups were performed using the Wilcoxon rank sum test. The correlation between two numerical variables was performed using Spearman's correlation coefficient. The R statistical language (version 4.3.0; R Foundation for Statistical Computing, Austria) was used for the statistical analysis. Statistical significance was set at the 0.05 level.

## 3 Results

**FIGURE 1:**
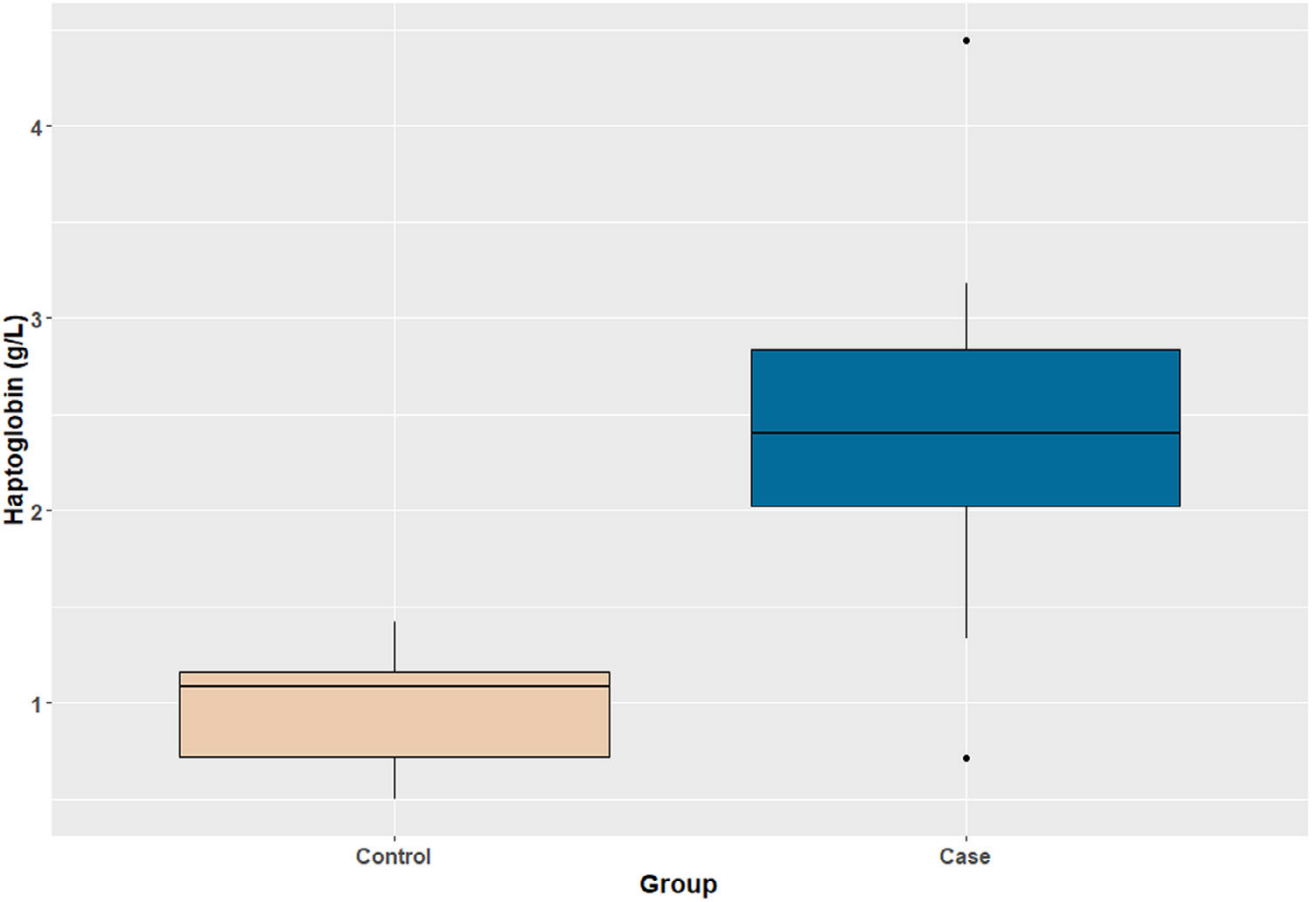
Comparisons of serum haptoglobin values between the control (healthy cats, *n* = 11) and case group (cats with gingivitis, *n* = 11). The coloured boxes represent the 25th to 75th percentiles of data; they are bisected by a line, which depicts the median value. Haptoglobin concentrations were significantly higher (*p *< 0.001) in the case group compared to the control group.

Based on the assumptions outlined above for the power analysis, the minimum required sample size was three animals per group. In total, 22 DSH cats (10 males and 12 females) were included in our study (11 in the control group and 11 in the case group). The median (range) age of cats was 5.0 (3.0–11.0) years. The median (range) TMPS‐G value for the case group was 0.815 (0.13–2.5). As per our inclusion criteria, TMPS‐G was equal to 0 in all cats included in the control group. The SAA was below the detection limit (0.4 mg/L) in all samples of the control group and in 10/11 samples of the case group. One sample from the case group had SAA of 0.5 mg/L. The median serum haptoglobin concentration was significantly higher (*p* = 0.001) in the case group [2.40 (0.72–4.44) g/L] compared to the control group [1.06 (0.50–1.42) g/L] (Figure 1). A minimal overlap was observed in haptoglobin between the two groups. Specifically, 2/11 (18.2%) cats with gingivitis had serum haptoglobin in the range of the healthy cats (Figure 1). Haptoglobin was above the reference interval (RI) (> 3 g/L) in 2/11 (18.2%) of cats with gingivitis, while it was within the RI in all healthy cats. A significant, moderate, positive correlation was found between haptoglobin and TMPS‐G (rho = 0.636, *p* = 0.040). Periodontal pocket depth was measured in all the cats included in this study, but it was within normal rates, so TMPS‐P was not used in statistical analysis.

## 4 Discussion

The results of this study indicate that cats with gingivitis have significantly higher concentrations of serum haptoglobin compared with healthy cats, suggesting the presence of an acute inflammatory reaction. Serum haptoglobin concentration was positively and moderately correlated with the severity of gingivitis. SAA was below the detection limit of the assay in all but one cat included in our study, and no further statistical analysis was possible.

Haptoglobin is an α_2_‐globulin and appears to have a higher molecular weight compared to SAA, although species‐specific studies are lacking currently (Petersen et al. 2004). It is mostly known for its ability to bind haemoglobin, but several other functions (e.g., bacteriostatic and immunomodulatory effects) have been attributed to haptoglobin (Eaton et al. 1982; El Ghmati et al. 1996). Sporadic studies have shown that haptoglobin can be increased during inflammatory conditions, with the highest values being reported in feline infectious peritonitis (Duthie et al. 1997; Gouffaux et al. 1975; Ottenjann et al. 2006).

In the present study, haptoglobin was significantly higher in cats with gingivitis compared with healthy cats, suggesting the presence of an acute‐phase reaction. This indicates that uncomplicated gingivitis can be associated with systemic inflammation, as has been suggested for periodontal disease. Although periodontal disease was initially attributed to the bacterial colonisation of the periodontal pocket, it is now believed that the host immune response to the bacterial products is of utmost importance (McFadden and Marretta 2013; Rossa and Kirkwood 2012). Specifically, it has been suggested that the destruction of endothelial cells together with the production of proinflammatory cytokines (such as IL‐1, IL‐6 and TNF‐α), among other mediators, increase the regional vascular porosity, which might allow bacteria and their products (e.g., endotoxins) and inflammatory mediators to enter the systemic circulation and cause an acute phase reaction (Wolf et al. 2005).

In a recent study, haptoglobin was found to be increased in cats with chronic gingivostomatitis (Polkowska et al. 2018). In that study, the increases in haptoglobin were more dramatic than the ones reported in our study. This can be explained by the differences in the severity between the two diseases. In our study, cats with gingivitis without clinical evidence of periodontitis or stomatitis were included as opposed to the previous study, which included cats with chronic gingivostomatitis (Polkowska et al. 2018).

Serum haptoglobin was positively and moderately correlated with the severity of gingivitis, as this was assessed using the TMPS‐G index (Harvey et al. 2008), and it was above the RI in 2 out of 11 cats included in our study. This indicates that serum haptoglobin might be a useful marker of feline gingivitis and its severity and that serum haptoglobin could possibly serve as a monitoring tool in these cats. Indeed, in cats with chronic gingivostomatitis, a significant decreasing trend in haptoglobin was noted during treatment, supporting its role in the monitoring of the disease (Polkowska et al. 2018). However, further studies are warranted to confirm serum haptoglobin as a monitoring tool for feline gingivitis. Given that TMPS‐P has not been extensively studied in cats, the authors believe it should not be used to assess advanced disease in this species at present. However, previous studies on TMPS‐P in dogs with advanced disease suggest that the grading system could be an effective and promising evaluation method.

SAA is a small protein with a molecular weight between 9 and 15 kDa, depending on the species (Cerón et al. 2005). It is normally found in complexes with lipoproteins and has several biological functions, such as transport of cholesterol from dying cells to hepatocytes (Liang and Sipe 1995), chemotactic effects on neutrophils, monocytes and lymphocytes (Badolato et al. 1994; Xu et al. 1995), and inhibition of platelet activation, just to name a few (Zimlichman et al. 1991). SAA has been found to increase in early stages of inflammation (Giordano et al. 2004; Kajikawa et al. 1999), but increases have also been reported with renal and hepatic diseases, diabetes mellitus and neoplasia (Sasaki et al. 2003). Unfortunately, all control cats and 10/11 cats with gingivitis had SAA values below the detection limit of the assay, which precluded further analysis. It would be interesting to explore if the development and introduction of more sensitive methods that could accurately measure SAA below the current limit of detection could detect changes in SAA in this disease. Nonetheless, it is also possible that SAA is inherently not a suitable biomarker for gingivitis in cats due to the localised and relatively mild nature of the inflammation, which may not trigger a systemic acute phase response detectable by circulating SAA concentration. This limitation highlights the need for continued exploration of more specific and sensitive diagnostic markers for feline gingival disease, such as IL‐1, IL‐6 or TNF‐α.

The small sample size should be mentioned as a limitation of the present study. This precluded any comparisons between cats that differ in disease severity. Additionally, SAA could not be measured in the vast majority of cats in this study due to the detection limit of the assay.

## 5 Conclusions

Feline gingivitis appears to be associated with increased serum haptoglobin as compared to healthy cats, suggesting the presence of an acute phase reaction. Serum haptoglobin was significantly correlated with the severity of gingivitis, and its role as a prognostic and monitoring tool should be further evaluated. However, due to the small sample size, it is difficult to draw conclusions about any differences in serum haptoglobin concentrations between cats with varying severities of gingivitis.

## Author Contributions

**Ioannis L. Oikonomidis**: investigation, writing – original draft, visualisation, writing – review and editing, formal analysis, data curation, methodology, validation. **Ioannis Kavarnos**: investigation, writing – review and editing. **Serafeim Papadimitriou**: investigation, writing – review and editing. **Jose Joaquin Ceron**: investigation, validation, methodology, writing – review and editing. **Maria Kouki**: investigation, writing – review and editing. **Katerina K. Adamama‐Moraitou**: investigation, writing – review and editing. **Nectarios Soubasis**: conceptualisation, investigation, writing – review and editing, supervision, methodology.

## Ethics Statement

The authors confirm that the ethical policies of the journal, as noted on the journal's author guidelines page, have been adhered to. No ethical approval was required, as all samples used in this study were residual materials from clinical cases.

## Conflicts of Interest

The authors declare no conflicts of interest.

## Peer Review

The peer review history for this article is available at https://www.webofscience.com/api/gateway/wos/peer‐review/10.1002/vms3.70606.

## Data Availability

The data are available from the authors upon request.
